# Gold Nanorod-Doped Acrylic Bone Cement: Mechanical Stability and Feasibility of Photothermal Therapy

**DOI:** 10.7759/cureus.105384

**Published:** 2026-03-17

**Authors:** Peter Bueide, Alexander Chong, Reese Anderson, Darin Ulness

**Affiliations:** 1 Orthopedic Surgery, Sanford Health, Fargo, USA; 2 Orthopedic Surgery, University of North Dakota School of Medicine and Health Sciences, Grand Forks, USA; 3 Department of Chemistry, Concordia College, Moorhead, USA

**Keywords:** bone cement, gold nanorods, nanomaterials, nanoparticles, photodynamic therapy (pdt), photothermal therapy, polymethylmethacrylate (pmma)

## Abstract

Purpose: Photothermal therapy (PTT) involves the conversion of electromagnetic radiation into heat to achieve targeted destruction of cells or tissue. Gold nanorods (GNRs) are commonly used nanoparticles in PTT due to their efficient photothermal conversion properties. The purposes of this biomechanical study were 1) to evaluate the in vitro feasibility of PTT using GNR-doped acrylic bone cement and 2) to assess the mechanical properties of the GNR-doped bone cement before and after PTT exposure.

Methods: Forty-eight polymethyl methacrylate (PMMA) bone cement tensile specimens were prepared, with groups representing GNR concentrations of 0, 10, 50, and 100 µg/mL (irradiated: six specimens/group; non-irradiated: six specimens/group). A 1064 nm laser with a fluence of 2.85 W/cm^2^ was used to irradiate the specimens. Heat generation and the time required to reach the target temperature (70°C on the non-irradiated surface) were recorded using two thermal imaging cameras. Mechanical tensile testing to failure was performed, and ultimate tensile strength (UTS) and elastic modulus (E) were subsequently measured for each sample.

Results: An increase in GNR concentration within PMMA samples resulted in faster and greater heat generation on the irradiated surface of the specimens, regardless of whether irradiation time or posterior (non-irradiated) surface temperature was held constant. No statistically significant differences in UTS or E were observed between irradiated and non-irradiated specimens at any GNR doped concentration (p > 0.05). However, a statistically significant difference in E was found when comparing the non-irradiated control group to both irradiated and non-irradiated GNR-doped samples. The magnitude of this reduction was less than 0.23 GPa, suggesting limited clinical relevance.

Conclusion: GNR-doped PMMA bone cement demonstrates significant potential as a platform for PTT, achieving temperatures well above those reported in the literature as effective for therapeutic purposes. Importantly, GNR doping PMMA does not produce any appreciable changes in UTS or E before or after PTT exposure.

Statement of clinical significance: The findings of this study contribute valuable insight to the limited body of evidence on GNR-doped PMMA. These results support the potential feasibility of using PTT through a novel delivery platform, which may ultimately be applied in the treatment of bone tumors and prosthetic joint infection. Future research should focus on elucidating the specific PTT parameters necessary for anti-tumor and bactericidal efficacy.

## Introduction

Gold nanorods (GNR), widely studied for their applications in photothermal therapy (PTT), have seen limited use in orthopedic surgery to date. Nevertheless, preclinical studies, including in vitro experiments and animal models, have demonstrated their potential in targeting osteosarcoma [1] and in ablating bacterial biofilms through PTT [2,3]. These findings suggest promising applications in managing some of the most challenging conditions in orthopedic practice, such as benign and malignant primary bone tumors, metastatic bone tumors, and prosthetic joint infections [4-8].

PTT involves the use of a photosensitizing agent capable of converting electromagnetic energy in the form of light into heat [9,10]. PTT is an attractive therapeutic approach due to its ability to induce minimally invasive target cell death, deep tissue penetration, and immediate therapeutic effect as demonstrated in various cancer models [1,2,11-14]. The thermal efficacy of PTT depends on several factors, including the photothermal conversion efficiency of the agent, nanoparticle concentration, and the power of the incident light source [15,16]. The available information in the literature raises an important question: can a PTT platform utilizing GNRs be leveraged in orthopedic surgery for targeted applications such as biofilm eradication and tumor ablation?

Polymethyl methacrylate (PMMA) bone cement is frequently used in both primary and revision arthroplasty, as well as for filling bony defects in the management of tumors. Given its widespread use and structural role, PMMA presents a promising potential carrier for nanoparticles to facilitate PTT. Historically, chemotherapeutic drugs or antibiotics have been incorporated into PMMA bone cement to reduce or prevent local tumor recurrence and infection [17-19]. However, the clinical efficacy of these approaches has been limited by the suboptimal and time-dependent elution profile of the embedded drugs [20-23]. To date, no studies have specifically explored the use of GNR-doped PMMA for PTT. Furthermore, it is unclear if adding GNRs to PMMA for PTT could negatively impact PMMA's mechanical properties.

The primary purpose of this study was to evaluate the in vitro feasibility of PTT using GNR-doped PMMA bone cement. Secondarily, we sought to assess the mechanical properties of the GNR-doped bone cement before and after PTT exposure. This study hypothesizes that GNR-doped PMMA will generate significantly more heat upon exposure to 1064 nm laser irradiation compared to a control group. Secondarily, we hypothesize there will be no measurable difference in mechanical properties between GNR-doped PMMA samples subjected to PTT and a non-PTT control group.

## Materials and methods

**Figure 1:**
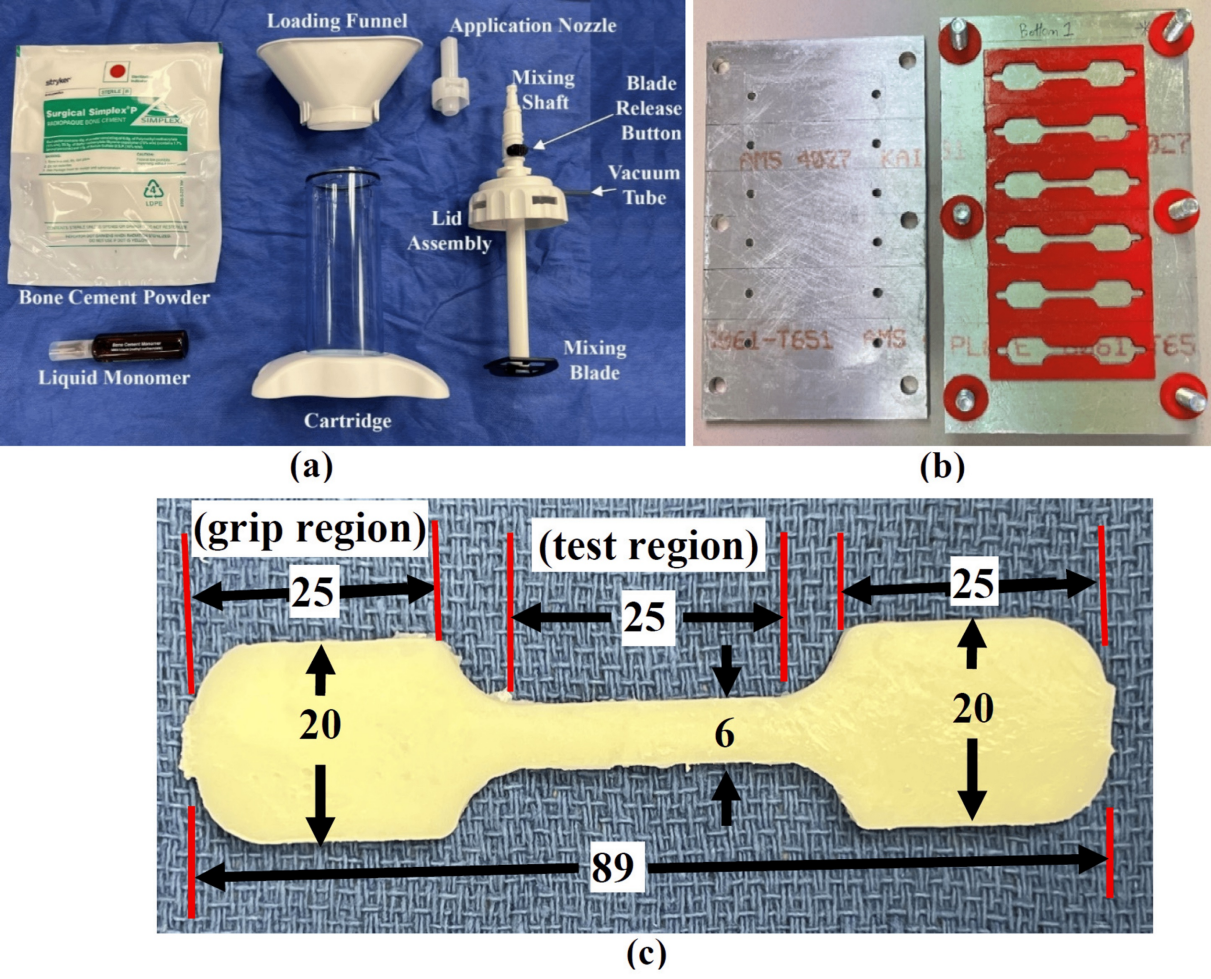
Polymethyl methacrylate (PMMA) bone cement materials and preparation setup.(a) Commercially available acrylic PMMA bone cement with advanced one-step cement mixing and delivery system, (b) bone cement sample mold for production of uniform tensile testing samples, and (c) tensile testing sample dimensions (mm).

This was a bench-top biomechanical study. The commercially available acrylic PMMA bone cement, Simplex^TM^ P (Stryker Howmedica Osteonics, Mahwah, NJ), was used in conjunction with an advanced one-step cement mixing and delivery system (Stryker Revolution Cement Mixing and Delivery System, Stryker Howmedica Osteonics, Mahwah, NJ) (Figure 1a). The PMMA bone cement consisted of a powder component and a liquid monomer component. The powder component was composed of 30.0 g of methyl methacrylate-styrene copolymer, 6.0 g of PMMA, and 4.0 g of barium sulfate. The liquid component was composed of 19.5 mL of methyl methacrylate, 0.5 mL of N-N dimethyl para-toluidine, and 1.5 mg of hydroquinone. GNRs (Nanopartz, Loveland, CO) had a diameter of 10 nm and a length of 67 nm, exhibiting a surface plasmon resonance of 1,064 nm (peak excitation wavelength). These GNRs were polystyrene-coated and suspended in a 7:3 mixture of 1-butanol and acetonitrile at a concentration of 30 µg/mL. A 1,064 nm continuous wave laser (Atomstack R30, Hong Kong, China) pulsed at 20 kHz with a time-averaged optical output power of 2 Watts (W) was used for excitation. Thermal data were recorded using two Flir One Edge Pro thermal cameras (Teledyne Flir, Wilsonville, Oregon). Each camera had a resolution of 160 x 120 pixels, a thermal accuracy of ±3ºC, and was configured with an emissivity setting of 0.95 and a temperature detection range of 0ºC to 400ºC. Both cameras were used to capture real-time samples’ surface temperature data during experimentation.

Four groups of PMMA bone cement samples were prepared based on concentrations used in the literature: bone cement without GNRs (control), bone cement with 33 µL of GNR solution (mass/mass concentration: 8.47 µg/g, estimated mass/volume concentration 10 µg/mL, GNR 10), bone cement with 165 µL of GNR solution (mass/mass concentrations 42.6 µg/g, estimated mass/volume concentration 50 µg/mL, GNR 50), and bone cement with 330 µL of GNR solution (mass/mass concentration 84.7 µg/g, estimated mass/volume concentration 100 µg/mL, GNR 100) [3,10,15]. The mass of the PMMA powder and liquid components was measured using an analytical balance (Ohaus, Pioneer, Parsippany, NJ) to determine the appropriate volume of GNR solution for each group. All procedures were performed in accordance with Occupational Safety and Health Administration (OSHA) universal precautions, and the manufacturer's standard handling protocols were followed. Sample mixing was carried out using the advanced one-step mixing system under the following standard protocol. An automated micropipette (Eppendorf, Hamburg, Germany) was used to transfer the specific volume of GNR solution of each group into the liquid monomer in a beaker. The GNR-monomer mixture was placed in an ultrasonic bath for five minutes under ambient conditions to ensure uniform dispersion of the nanoparticles. The PMMA powder was then added to the mixing cartridge, followed by the GNR-monomer solution. The lid assembly was secured, and the cement samples were subjected to a vacuum pressure of 508-559 mmHg. The mixing shaft of the lid assembly was connected to a surgical rotary power tool set to the “ream” mode and operated for 90 seconds. During mixing, the shaft was moved up and down to ensure homogeneity of the cement mixture. All samples were prepared at standard operating room temperature (18-19°C). The mixing methods utilized were chosen to mimic dispersion in the clinical environment using GNRs as an additive to PMMA. 

A custom-designed mold was used to produce six standard tensile samples simultaneously (Figure 1b). American Society for Testing and Materials (ASTM) D638 IV dimensions were utilized, with a shortened clamp length. The mixed cement from each group was transferred into the mold using a cement injection gun set to the “fill” mode. To minimize air entrapment, special care was taken to avoid layering of cement during injection. After injection, the samples were allowed to solidify in the mold under pressure for one hour, then removed and allowed to fully cure for at least 24 hours prior to testing. Each experimental group included 12 tensile specimens, subdivided as six irradiated specimens and six non-irradiated specimens. Figure 1c illustrates the detailed geometry of each tensile cement sample, with a uniform thickness of 3 mm. All samples underwent visual inspection for surface defects and any powder clots, and any visibly flawed samples were excluded from testing. The cross-sectional area of the test regions for each sample was measured using a digital caliper (General Tools & Instruments, Secaucus, NJ) prior to mechanical testing. In addition, radiographs were obtained for all samples to detect any internal defects not visible on external inspection. The mechanical experimental setup and protocol used in this study were consistent with previously published methods [24-26].

Laser preparation

**Figure 2:**
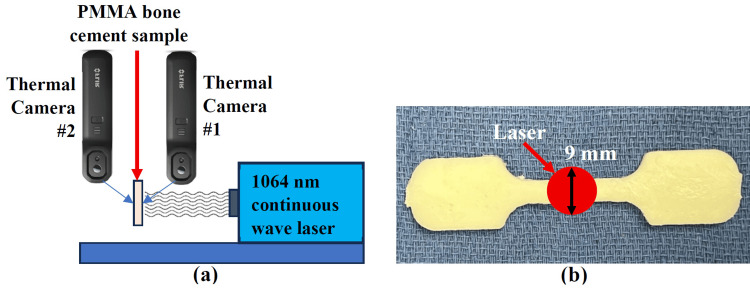
Photothermal irradiation experimental setup. (a) Sketch drawing of the laser and thermal camera orientations relative to the test sample, and (b) depiction of 9 mm diameter laser irradiation on the test region of the tensile specimens.

The sample stage was positioned 354 mm from the laser aperture. A power meter (Gentec PSV-3103, Quebec City, Canada) was used to verify the laser output at the aperture, confirming a power of 2 W. An iris diaphragm with a 7 mm diameter opening was placed 173 mm from the laser aperture to adjust the spot size on the sample surface. After placement of the iris, the transmitted power measured was 1.84 W through the aperture. To characterize the laser beam profile at the sample location, a high-sensitivity power meter (S140C, Thorlabs, Newton, New Jersey) was used. The beam was profiled at the point of maximum intensity, with measurements taken at 0.5 mm increments along the horizontal axis. Assuming a Gaussian beam distribution, the 1/e2 radius (corresponding to 13.5% intensity level) was determined to be 4.5 mm, resulting in a beam diameter of 9 mm at the sample surface. This configuration yielded a laser fluence of 2.85 W/cm2 at the sample. One thermal camera was placed 110 mm posterior and oblique to the center of the sample’s test region, while the second thermal camera was positioned 160 mm anterior and oblique to the test region (Figure 2). All experiments were conducted at ambient room temperature in the laboratory, ranging between 19.8°C and 19.9°C, with standard laboratory room air turnover maintained throughout the experiments.

Photothermal irradiation protocol

All photothermal experiments were performed by irradiating the tensile test region of each specimen after securing it in a custom-designed specimen holder. This holder ensured consistent and reproducible positioning of each sample relative to the laser beam (Figure 2). Laser irradiation was applied until the maximum temperature on the posterior (non-irradiated) surface of the specimen reached 70°C. The target temperature selected was based on previous studies demonstrating effective GNR PTT against microorganisms (bactericidal and antibiofilm) [3]. Once the posterior surface reached 70°C, the laser was immediately turned off, and the time required to reach this temperature was recorded. For the control group (no GNRs), the samples were irradiated for a duration of 120 seconds, as the 70°C threshold was not achieved. This 120-second period was chosen to match the longest irradiation duration observed among GNR-doped groups.

The maximum surface temperatures on both the anterior (irradiated) and posterior (non-irradiated) sides were recorded at the 15-second mark, which was the shortest irradiation time among all samples. These early temperature readings were used to assess the rate of heat generation and to control for time-dependent effects across different experimental groups.

Photothermal efficacy

**Figure 3:**
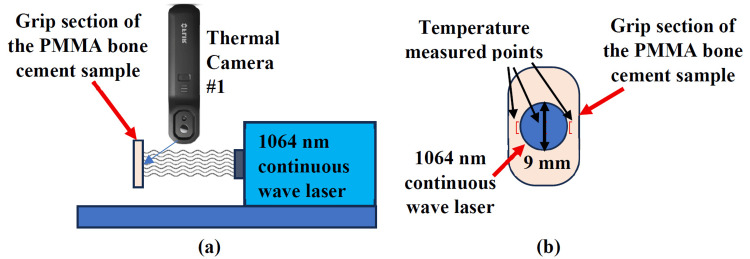
Photothermal efficacy experimental setup. (a) Sketch drawing of the experimental setup and (b) 9 mm diameter laser at the center of the PMMA bone cement sample.

Another custom specimen holding jig with a 10 mm cutout width was used. This setup ensured that the grip section of each sample could be positioned consistently relative to the laser beam. The cutout was centered on the laser beam using a power meter (Gentec PSV-3103, Quebec City, Canada), confirming that 1.84 W of laser power (corresponding to 2.85 W/cm^2^) passed through the opening. One randomly selected sample from each PMMA group was used for this evaluation (Figure 3). The laser was activated and maintained until the irradiated surface reached a steady state temperature. The irradiated surface temperatures were recorded using thermal camera #1, capturing thermal images every 10 seconds at three predefined points: the center of the laser beam, 5 mm lateral to the center beam (right edge of the beam), and 5 mm medial to the center beam (left edge of the beam).

Mechanical tensile testing

All samples underwent mechanical tensile testing to failure. A servo-hydraulic materials testing system (Model 8874; Instron, Norwood, MA) equipped with 25 kN load cells was used to perform load-to-failure tensile testing for each sample group. Each specimen was subjected to uniaxial tensile loading, beginning from 0 N and continuing until complete structural failure, at a constant stroke rate of 2.54 mm/min. Load and deflection data were recorded at a sampling frequency of 100 Hz. The ultimate tensile strength (UTS) and the tensile elastic modulus (E) were then determined. This mechanical test was performed in air at a room temperature of 21°C.

Statistical analysis

Descriptive statistics of the mean, standard deviation, and range were calculated for all measured variables. To assess differences between groups, one-way analysis of variance (ANOVA) with the least significant difference (LSD) multiple comparisons post-hoc test method was utilized. These analyses were used to evaluate the effects of experimental groupings on key parameters, including UTS, E, sample surface temperature, and irradiation time. All statistical analyses were conducted using IBM SPSS Statistics software (version 24.0; IBM Corporation, Armonk, NY), and a P value less than 0.05 was considered statistically significant.

## Results

Photothermal irradiation of tensile specimens

**Figure 4:**
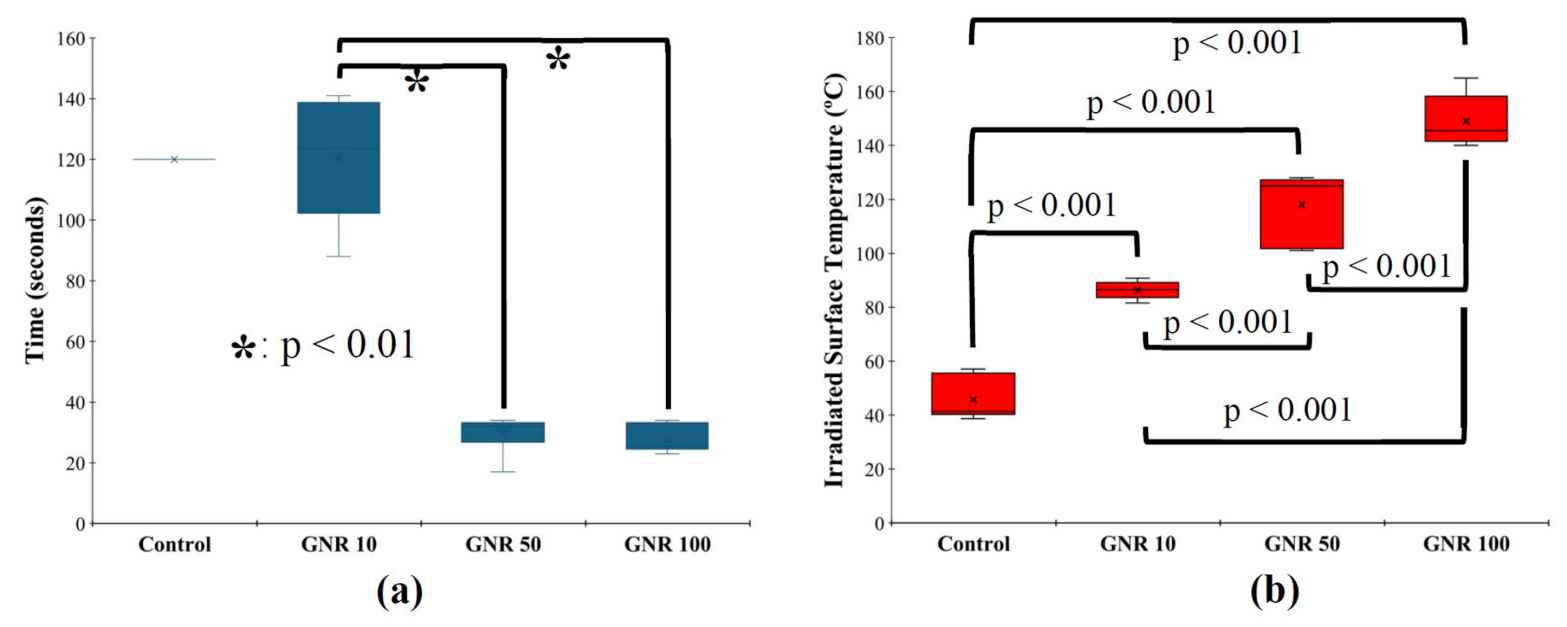
Mean time and mean surface temperature for irradiated specimens when the backside of the specimen reached the endpoint temperature of 70ºC. (a) Mean time and (b) mean maximum irradiated (front) surface temperature. Image created by the authors with Microsoft Office Suite (Microsoft Corp., USA)

The mean time for posterior temperature to reach 70°C was 120 ± 20 seconds for the GNR 10 group, 30 ± 6 seconds for the GNR 50 group, and 28 ± 5 seconds for the GNR 100 group. By contrast, the control group (no GNRs) reached a maximum posterior (non-irradiated) temperature of only 40°C, even after 120 seconds of continuous laser irradiation. Statistical analysis revealed a significant reduction in time required to reach the target temperature of 70ºC in both the GNR 50 and GNR 100 groups compared to the GNR 10 group (Figure 4a, p < 0.05).

At the moment when the posterior (non-irradiated) surface reached 70°C, the corresponding anterior (irradiated) surface temperatures were 86 ± 3°C for the GNR 10 group, 118 ± 13°C for the GNR 50 group, and 149 ± 10°C for the GNR 100 group. The control group reached an anterior surface temperature of 46 ± 8°C. Statistical analysis demonstrated a significant difference in the mean maximum irradiated surface temperature between all groups with p < 0.001 (Figure 4b).

**Figure 5:**
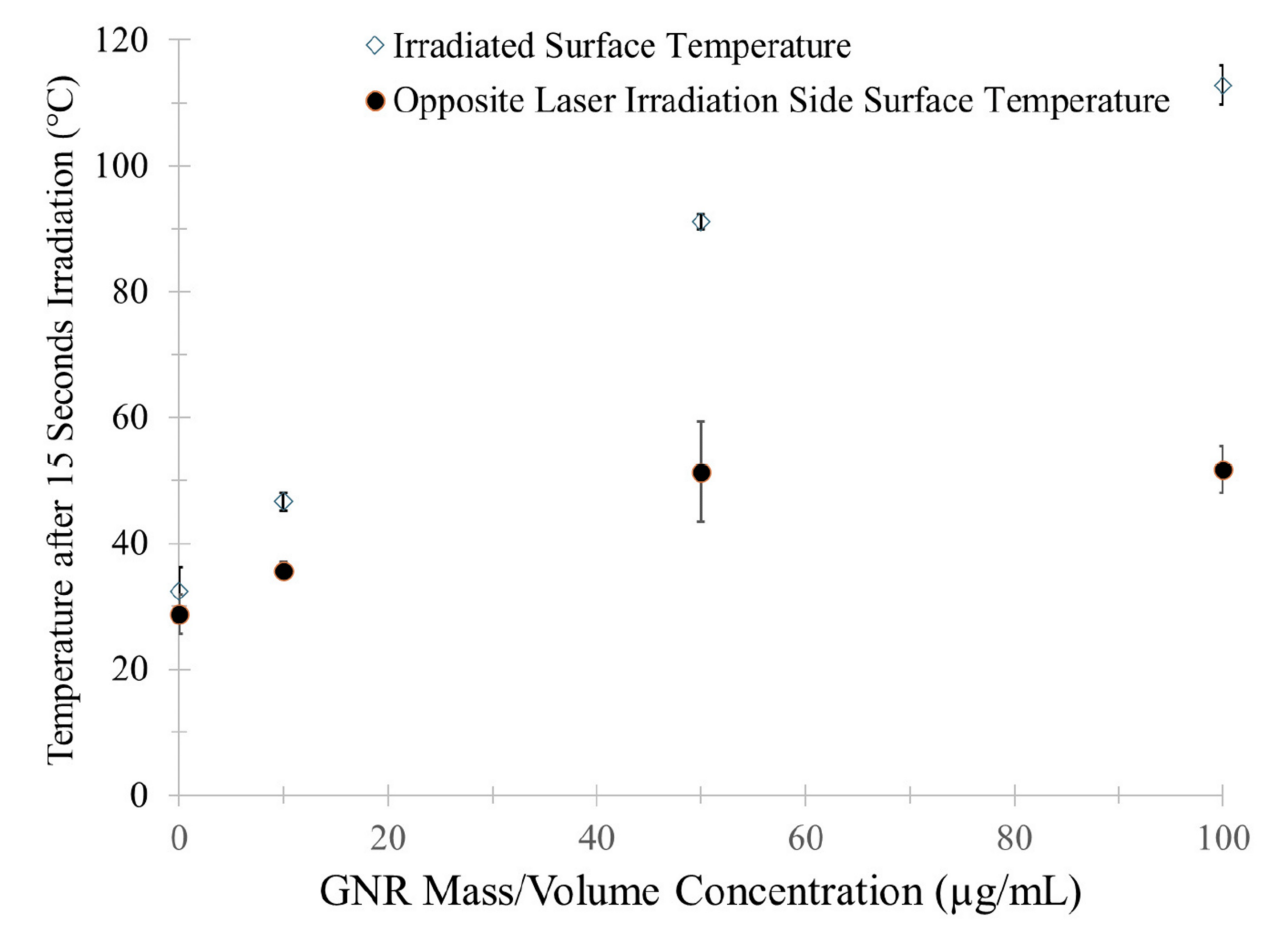
Mean surface temperature on irradiated surface and opposite surface of the specimens after exposure to 2.85 W/cm2 of 1064 nm laser irradiation for 15 seconds. Image created by the authors with Microsoft Office Suite (Microsoft Corp., USA)

At 15 seconds of laser irradiation, the mean irradiated surface temperatures were 32 ± 4ºC for the control group, 47 ± 1ºC for the GNR 10 group, 91 ± 1ºC for the GNR 50 group, and 113 ± 3ºC for the GNR 100 group. The mean posterior (non-irradiated) surface temperatures were 29 ± 3ºC for the control group, 36 ± 1ºC for the GNR 10 group, 51 ± 8ºC for the GNR 50 group, and 52 ± 4ºC for the GNR 100 group (Figure 5).

Photothermal efficacy

**Figure 6:**
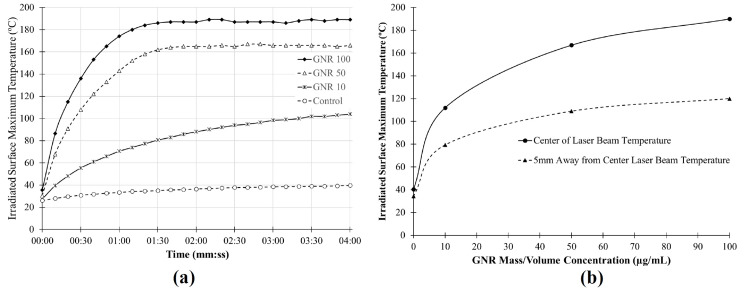
Specimen surface temperature with photothermal irradiation. (a) Irradiated surface maximum temperature over time, and (b) maximum temperature reached for each concentration of GNR-doped PMMA at the center and edge of the beam (5 mm away from the center of the laser beam) on the irradiated surface. GNR: gold nanorod, PMMA: polymethyl methacrylate Image created by the authors with Microsoft Office Suite (Microsoft Corp., USA)

All specimens exhibited an initial phase of rapid temperature increase, followed by a plateau indicating that a steady state temperature had been reached (Figure 6a). As the GNR concentration increased, both the maximum temperature and the temperature gradient across the laser beam profile also increased (Figure 6b). This reflects the enhanced photothermal conversion efficiency at higher nanoparticle concentrations and a more pronounced thermal localization effect near the beam center.

Mechanical tensile testing

**Figure 7:**
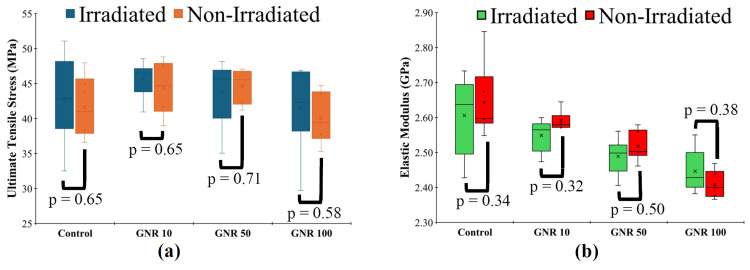
Non-irradiated versus irradiated polymethyl methacrylate (PMMA) mechanical properties comparison. Image created by the authors with Microsoft Office Suite (Microsoft Corp., USA)

There were no significant differences observed in UTS or E between the irradiated and non-irradiated specimens within each GNR doped concentration group (Figure 7). In addition, the UTS values at all GNR concentrations, regardless of laser irradiation, did not differ significantly from those of the non-irradiated control group (Table 1). However, a statistically significant difference in E was detected between the non-irradiated control group and both irradiated and non-irradiated GNR-doped samples. The magnitude of this difference was less than 0.23 GPa.

**Table 1:** Statistical analysis for mechanical properties comparison between groups. *statistically significant

	Ultimate tensile stress		Elastic modulus	
	Mean ± SD (MPa)	p-value	Mean ± SD (GPa)	p-value
Control (non-irradiated)	41.6 ± 4.5	Reference	2.64 ± 0.11	Reference
GNR 10 (non-irradiated)	44.4 ± 4.1	0.296	2.59 ± 0.03	0.159
GNR 50 (non-irradiated)	44.7 ± 2.5	0.243	2.52 ± 0.04	0.002*
GNR 100 (non-irradiated)	40.0 ± 3.5	0.544	2.41 ± 0.04	<0.001*
GNR 10 (irradiated)	45.6 ± 2.6	0.137	2.55 ± 0.05	0.019*
GNR 50 (irradiated)	43.8 ± 4.8	0.421	2.49 ± 0.05	<0.001*
GNR 100 (irradiated)	41.5 ± 6.3	0.954	2.45 ± 0.06	<0.001*

## Discussion

The present mechanical study is the first to demonstrate the in vitro feasibility of incorporating GNR into acrylic bone cement for PTT and to compare the mechanical properties of varying concentrations of GNR-doped PMMA before and after PTT. The key finding is that GNR-doped PMMA demonstrates heating under 1064 nm laser irradiation that may serve as a platform for PTT without compromising tensile integrity.

PTT has been extensively explored in the biomedical field. In this study, GNRs measuring 67 x 10 nm were utilized, enabling spectral overlap with the 1064 nm wavelength emitted by the laser. This alignment corresponds to the transverse plasmon resonance of the GNRs, facilitating efficient photothermal conversion. Near-infrared wavelengths (1064 nm) have been shown to allow greater tissue penetration, making them particularly suitable for clinical application [10,27]. As hypothesized, the results demonstrated that increasing the concentration of GNRs within the PMMA matrix led to greater heat generation under 1064 nm laser irradiation. Similarly, higher GNR concentrations resulted in greater temperature gradients, both across the laser beam profile (center and edge) and between the anterior (irradiated) and posterior (non-irradiated) surfaces of the specimens. This thermal behavior may be attributed to a combination of the Gaussian laser beam profile creating a central concentration of power on the irradiated surface and the enhanced light absorbance on the irradiated face at higher concentrations of GNR, limiting the photothermal heating of GNRs further along the path of the laser in the samples. Ultimately, heating to a controlled target temperature is possible in future studies using a specific GNR concentration and laser fluence combination [15]. While we obtained a plateau temperature of over 180ºC in the GNR100 sample, this was relative to the fluence of 2.85 W/cm^2^, and we would not recommend high temperatures that could damage adjacent native tissues in future studies. Similarly, irradiated front side temperatures of tensile specimens obtained during PTT exposure highlight the need for a controlled GNR concentration and laser fluence combination to heat to a specific, and safe, target plateau temperature in future work. 

The present study secondarily demonstrated that the addition of GNRs does not substantially alter the tensile properties of PMMA. There was also no effect on tensile properties from PTT exposure under the parameters tested. At all tested GNR concentrations, there was no statistically significant difference in UTS or E between irradiated (PTT-exposed) and non-irradiated samples. This was observed despite the surface temperature reaching levels two to four times higher than those required to destroy targeted cells [3,9,28]. These findings suggest that the PTT parameters tested, including irradiation time and maximum surface temperature reached, did not induce structural changes in PMMA that compromise mechanical strength. Furthermore, when compared to the non-GNR-doped, non-irradiated control group, no significant differences in UTS were detected across any GNR-doped concentration, regardless of irradiation exposure. This demonstrates that GNR-doping at the tested concentrations does not induce a significant change in UTS. However, a significant decrease in E was observed in GNR-doped samples compared to the non-GNR-doped, non-irradiated controls. This reduction in E, which trended down with increasing GNR concentration, reached a maximum difference of 0.23 GPa. This suggests an effect of GNR-doping concentration on E. However, the magnitude of change, 0.23 GPa, is of questionable clinical significance. 

The findings of this study are consistent with previous reports in the literature [29,30]. Swieczko-Zurek and colleagues investigated the influence of various nanometals, including silver (grain size 40 nm), copper (10-30 nm and 70-100 nm), silver with copper (90 nm), and nickel (10-30 nm), incorporated into PMMA bone cement at a concentration of 1.5 weight% [29]. They concluded that these nanometal particles provided long-term antibacterial resistance without significantly compromising compression strength. Similarly, Russo et al. demonstrated that 10-20 nm gold nanoparticles did not adversely affect compressive modulus or maximum tolerated stress when incorporated into PMMA at concentrations below 1 weight% [30]. In comparison, the highest GNR concentration used in the present study was over 100 times more dilute than those used in previous investigations. However, it is important to note that none of these earlier studies evaluated the effect of PTT on the mechanical properties of PMMA composites.

This study has several limitations that warrant consideration. First, this is a benchtop study that has not proven bactericidal or anti-tumor applications. However, this was beyond the scope of the study’s primary objective, which was to assess the feasibility of GNR-doped bone cement for a PTT platform. Second, it was impossible to control for GNR concentration, temperature, time of exposure, and laser fluence simultaneously. Future work needs to elucidate the mechanical properties of GNR-doped PMMA after exposure to these specific PTT parameters used for bactericidal or anti-tumor purposes. This may also include different durations of PTT exposure and multiple irradiation cycles. Third, GNR dispersion within samples was not assessed to mimic the clinical process of adding GNR to PMMA with simple mixing. However, given consistency in heating data and mechanical testing at each concentration, we infer relative homogeneity of samples, but further confirmatory tests would be required. Fourth, only a single load-to-failure tensile test was performed. More clinically relevant mechanical tests, such as four-point bending, compression, or fatigue testing under cyclic loading, may better represent the mechanical behavior in clinical applications. Finally, only a single type of bone cement was evaluated, which limits the generalizability of the findings to other commercially available formulations. Further research is needed to validate the findings of this study and to determine the optimal parameters for effectively targeting bacterial and tumor cells using GNR-doped PMMA.

## Conclusions

GNR-doped PMMA demonstrates potential as a platform for PTT, achieving temperatures well above those reported in the literature as effective for therapeutic purposes. Importantly, it does not produce any appreciable changes in UTS or E before or after PTT exposure under the parameters tested. Further work is necessary to characterize the efficacy of the platform in anti-bacterial and tumor applications that may be beneficial in clinical applications.
